# Clinical characteristics and early prediction of mortality risk in patients with acute organophosphate poisoning-induced shock

**DOI:** 10.3389/fmed.2022.990934

**Published:** 2023-01-11

**Authors:** Bing Xu, Weijia Zeng, Feng Chen, Gui Lin, Mengjie Wang, Jie Ding, Ye Hong, Jun Ke, Xiaoping Wang, Xiuling Shang

**Affiliations:** ^1^Department of Emergency, Shengli Clinical Medical College of Fujian Medical University, Fujian Provincial Hospital, Fujian Emergency Medical Center, Fujian Provincial Key Laboratory of Emergency Medicine, Fuzhou, China; ^2^The Third Department of Critical Care Medicine, Shengli Clinical Medical College of Fujian Medical University, Fujian Provincial Hospital, Fujian Provincial Center for Critical Care Medicine, Fujian Provincial Key Laboratory of Critical Care Medicine, Fuzhou, China

**Keywords:** acute poisoning, organophosphate insecticides, hypotension, shock, prognosis

## Abstract

**Objective:**

To further get insights of clinical characteristics of acute organophosphate poisoning-induced shock, investigate the relationship between shock and prognosis, and screen risk indicators for prognosis.

**Methods:**

A total of 73 patients with acute organophosphate poisoning admitted to our hospital between January 2014 and December 2021 were enrolled in this retrospective study. Patients were divided into the shock group and the non-shock group. The pH value of blood, arterial blood carbon dioxide partial pressure (PaCO_2_), arterial partial pressure of oxygen (PaO_2_), base excess (BE), lactic acid (Lac), serum albumin (ALB), total bilirubin (TBIL), alanine aminotransferase (ALT), serum creatinine (Cr), serum potassium (K), serum calcium (Ca), serum sodium (Na), blood chloride (Cl), serum troponin I (cTNI), brain natriuretic peptide (BNP), white blood cell count (WBC), hemoglobin (HGB), platelet count (PLT), and other clinical indicators of patients were recorded. Incidence of shock, time of shock onset, and outcomes of patients were also recorded. Cox proportional hazards regression models were performed for analysis.

**Results:**

The incidence of organophosphate poisoning-induced shock was 30.1% (22/73), and 72.7% of shock patients developed shock blood pressure within 6 h. The levels of blood lactate, ALT, Cr, cTNI, BNP, and Cl in the shock group were significantly higher than those in the non-shock group, while the level of Ca and pH value was significantly lower than that in the non-shock group (all *p* < 0.05). Moreover, compared with patients without shock (2.0%), the mortality rate was significantly increased in patients with shock (36.4%), which was supported by the results from adjusted Cox proportional hazards regression model. We found that shock and elevated serum creatinine were associated with increased risk of death in patients with organophosphate poisoning (shock: HR, 10.9; 95% CI 1.2–96.3; elevated serum creatinine: HR, 1.0, 95% CI 1.0–1.0).

**Conclusion:**

This study indicated the association between elevated serum creatinine and increased mortality rates in patients with organophosphate poisoning, highlighting the importance of the comprehensive management of shock, especially the control of renal function, in these poisoning patients.

## Introduction

Organophosphate poisoning is the most common pesticide poisoning in the emergency department, with high morbidity and mortality. According to recent data, 3 million patients were poisoned, and at least 250,000 were killed yearly by organophosphorus poisoning (1, 2). The mortality rate of organophosphate poisoning is 3–40% in China (3). Although the application of routine therapeutic strategies, including atropine, oxime, and hemoperfusion, the success rate of their resuscitation still varies widely. Respiratory failure, severe coma, and shock are the main causes of organophosphate poisoning deaths (4, 5). With the widespread use of ventilators, respiratory failure has been effectively treated, while clinical diagnosis and treatment of organophosphate poisoning-induced shock are still challenging. Pannu et al. (6) reported an incidence of hypotension (initial systolic blood pressure <90 mmHg and mean arterial pressure <70 mmHg) of 13.5% in patients with organophosphate poisoning. They also found that hypotension was not associated with death. Thakur et al. (7) reported an incidence of hypotension on admission or within 24 h of admission (after poisoning) of 37.9%; patients with hypotension had lower Glasgow Coma Scale(GCS) scores, longer hospital stays, higher rates of (Intensive Care Unit) ICU admission, and higher rates of septic shock and aspiration pneumonia. Yu et al. (8) reported that the incidence of shock after organophosphate poisoning was 14.1%, and shock was an independent risk factor for death in these patients. However, the detailed definition of shock was not clarified in the study. In addition, the pathophysiological mechanism of organophosphate poisoning-induced shock is complex. Various types of shock, such as hypovolemic shock, cardiogenic shock, and distributive shock, can occur simultaneously, which leads to difficulty in clinical treatment. In the past, increasing the dose of atropine was believed to be effective in treating post-poisoning shock (9). However, Davies et al. (10) found that rapidly increasing the dose of atropine to 200 mg after admission could not rescue the refractory shock for patients with severe post-poisoning shock. According to the Consensus on circulatory shock and hemodynamic monitoring reported by the European Society of Intensive Care Medicine in 2014 (11), patients with decreased blood pressure, changes in consciousness, cold limbs, decreased urine output, and other signs of shock accompanied by an increase in blood lactate >2.0 mmol/L were diagnosed with shock. However, organophosphate poisoning with muscarinic symptoms and organophosphorus central nervous system damage differs from septic shock. Poisoning symptoms include clammy limbs, whole-body sweating, and disturbance of consciousness, which affects the identification of shock signs. At the same time, organophosphate poisoning-induced shock often occurs in the acute stage of poisoning within 24 h (1). Thus, the aim of this study was to clarify the clinical characteristics of acute organophosphate poisoning-induced shock, evaluate the relationship between shock and prognosis, and screen for risk indicators that affect prognosis.

## Materials and methods

### Patients

Patients with acute organophosphate poisoning were admitted to the emergency ward and intensive care unit of Fujian Provincial Hospital and Fujian Provincial South Hospital from January 2014 to December 2021. Inclusion criteria were: (1) age >18 years old; (2) those who met the criteria for organophosphate poisoning, i.e., with a clear history of exposure to organophosphorus poisons, muscarinic symptoms, such as miosis, hyperhidrosis, diarrhea, emesis and bronchorrhea, and nicotine-like symptoms, such as weakness and muscle fasciculation, which could be significantly improved by atropine treatment. Exclusion criteria were the following: (1) patients with malignant tumor or pregnancy; (2) patients who consulted the hospital >24 h after poisoning; (3) patients who gave up treatment within 24 h and were automatically discharged from the hospital; (4) patients with incomplete clinical and imaging data.

This study was approved by the Ethics Committee of Fujian Provincial Hospital (Approval number: K2021-12-069).

### Treatment for poisoning

The admitted patients were routinely treated with systemic scrubbing, gastric lavage, catharsis, and other methods to remove toxins, and intravenous injection of atropine. Then, continuous intramuscular injection of different doses of penehyclidine combined with oxime was given according to the severity. Patients with severe illness received hemoperfusion. Patients with respiratory failure were intubated and connected to a ventilator to maintain oxygenation. Patients with cardiac arrest received cardiopulmonary resuscitation and those complicated by acute renal failure or severe acidosis were treated with continuous renal replacement therapy (CRRT).

### Diagnosis and treatment of organophosphorus poisoning-induced shock

Patients with systolic blood pressure <90 mmHg, or mean arterial pressure <65 mmHg and arterial blood lactate >2 mmol/L, or norepinephrine who needed to maintain blood pressure within 24 h after poisoning were diagnosed with poisoning-induced shock and divided into two groups: the shock group and the non-shock group. For shock patients, the routine treatment of the Department of Critical Care Medicine of the Provincial Hospital was performed. In addition to active fluid replacement, norepinephrine was the first choice to maintain blood pressure, and if necessary, the combination of norepinephrine with dopamine was given to increase the blood pressure.

### Clinical data

The general information of patients from the electronic medical record system of Fujian Provincial Hospital and Fujian Provincial Hospital South Hospital, including gender, age, type of poison, poisoning dose, time after poisoning to our hospital, time of shock, use of intubation, information on cardiac arrest (if any). The pH value of blood, arterial blood carbon dioxide partial pressure (PaCO_2_), arterial partial pressure of oxygen (PaO_2_), base excess (BE), lactic acid (Lac), serum albumin (ALB), total bilirubin (TBIL), alanine aminotransferase (ALT), serum creatinine (Cr), serum potassium (K), serum calcium (Ca), serum sodium (Na), blood chloride (Cl), serum troponin I (cTNI), brain natriuretic peptide (BNP), white blood cell count (WBC), hemoglobin (HGB), platelet count (PLT), and other clinical indicators of patients were recorded. Data of hospital stay, total hospitalization costs, and the time of death (if any) were also included. Thirty-day mortality in the hospital was analyzed as a prognostic indicator, and death time was recorded.

### Statistical methods

SPSS 26.0 statistical software was used for the statistical analysis of the data. The measurement data with normal distribution were expressed as $\bar{x}\pm$s, and the independent sample t-test was used to compare the two groups. The data without normal distribution were expressed as M (P25, P75), and the Wilcoxon rank-sum test was used to compare the two groups. The categorical data were expressed as the case (%), and the chi-square test was used to compare the two groups. The 30-day mortality in the hospital was analyzed with death as the dependent variable. The Cox proportional hazards regression model was performed for univariate regression analysis. First, independent variables with *p* < 0.1 were screened out. Then, Cox multivariate regression analysis was performed according to the stepwise scheme. Kaplan–Meier survival analysis was performed using the Log-Rank test. A *p* < 0.05 was considered statistically significant.

## Results

### General clinical data of patients with organophosphorus poisoning-induced shock

**FIGURE 1:**
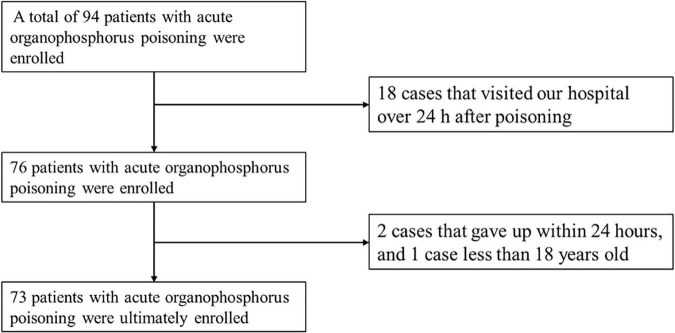
Eligibility criteria for study cohort participants.

**FIGURE 2:**
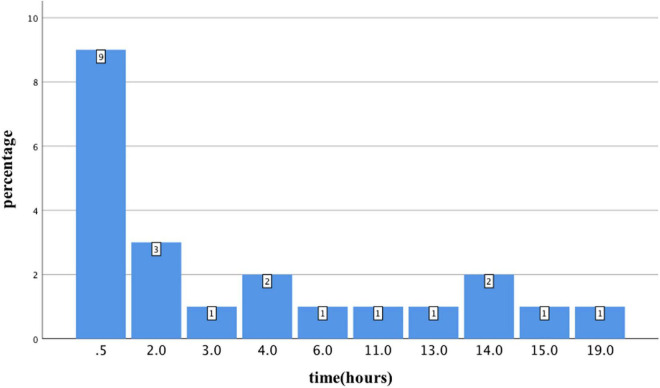
Histogram of the occurrence time of organophosphorus poisoning-induced shock.

From January 2014 to December 2021, 94 patients with acute organophosphorus poisoning were consecutively enrolled. After excluding 18 cases that visited our hospital over 24 h after poisoning, two cases that gave up within 24 h, and one case <18 years old, 73 cases with acute organophosphate poisoning were enrolled (Figure 1). The mean age was 50.1 ± 14.9 years old, with 31 males (43.5%) and 42 females (56.5%). There were 22 cases with shock, accounting for an incidence of 30.1%. Besides, 9 cases (40.9%) developed shock blood pressure at the time of consultation, 72.7% of cases developed shock blood pressure within 6 h of onset, and 6 cases (27.3%) developed shock blood pressure after 6 h. One case developed shock blood pressure after 19 h (Figure 2).

There was no significant difference in age, gender, time of visiting our hospital, and the distribution of underlying diseases between the two groups (*p* > 0.05). Dichlorvos was the most common organophosphorus poison, seen in 34 cases (46.6%). The proportion of dichlorvos poisoning in the shock group was significantly higher than in the non-shock group (68.2 vs. 37.3%, *p* = 0.015). The poisoning dose of dichlorvos in the shock group was significantly higher than that in the non-shock group (216.2 ± 124.0 vs. 172.3 ± 127.7, *p* = 0.008) (Table 1).

**TABLE 1:** General clinical data of patients with organophosphorus poisoning-induced shock.

	All patients (*n* = 73)	Shock group (*n* = 22)	Non-shock group (*n* = 51)	*P*-value
Age (year, $\bar{x}\pm$s)	50.1 ± 14.9	49.6 ± 13.8	50.4 ± 15.4	0.827
Male, *n* (%)	31 (42.5%)	13 (59.1%)	18 (35.3%)	0.059
**Types of poison, *n* (%)**				
Dichlorvos	34 (46.6%)	15 (68.2%)	19 (37.3%)	0.015
Dimethoate	13 (17.8%)	5 (22.7%)	8 (15.70%)	0.698
Methamidophos	8 (11.0%)	1 (4.5%)	7 (13.7%)	0.457
Other	16 (21.9%)	1 (4.5%)	15 (29.4%)	0.041
Toxic dose (ml, $\bar{x}\pm$s)	189.4 ± 130.0	228.3 ± 130.3	172.3 ± 127.7	0.090
Dose of dichlorvos (ml, $\bar{x}\pm$s)	156.5 ± 110.0	216.2 ± 124.0	108.1 ± 68.6	0.008
Time of admission [h M(P25, P75)]	5.0 (4.0–8.0)	5.0 (4.0–8.5)	5.0 (4.0–8.0)	0.961
**Comorbidities**				
Hypertension, *n* (%)	12 (16.4%)	2 (9.1%)	10 (19.6%)	0.442
Diabetes mellitus, *n* (%)	4 (5.5%)	0 (0)	4 (7.8%)	0.308
Mood disorder, *n* (%)	8 (11.0%)	1 (4.5%)	7 (13.7%)	0.457
Coronary heart disease, *n* (%)	2 (2.7%)	1 (4.5%)	1 (2.0%)	0.515

### Shock and prognosis

A total of 90.9% of the cases in the shock group required endotracheal intubation, while 33.3% in the non-shock group required endotracheal intubation. The levels of ALT, Cr, cTNI, BNP, and Cl in the shock group were significantly higher than those in the non-shock group, while the level of Ca was significantly lower than that in the non-shock group (all *p* < 0.05). Besides, the patients in the shock group had significantly lower pH and higher blood lactate (*p* < 0.05) (Table 2).

**TABLE 2:** Comparison of clinical indicators between shock group and no shock group.

	All patients (*n* = 73)	Shock group (*n* = 22)	Non-shock group (*n* = 51)	*P*-value
Intubation, *n* (%)	37 (50.7%)	20 (90.9%)	17 (33.3%)	<0.001
Cardiac arrest, *n* (%)	5 (6.8%)	5 (22.7%)	0 (0.0%)	0.002
PH ($\bar{x}\pm$s)	7.287 ± 0.155	7.173 ± 0.168	7.347 ± 0.108	<0.001
PaCO_2_ (mmHg, $\bar{x}\pm$s)	39.2 ± 15.7	41.8 ± 21.2	37.9 ± 12.3	0.546
PaO_2_ (mmHg, $\bar{x}\pm$s)	114.5 ± 82.9	116.6 ± 117.6	113.4 ± 59.1	0.098
BE (mmol/L, $\bar{x}\pm$s)	−8.02 ± 6.52	−12.89 ± 5.97	−5.77 ± 5.49	<0.001
Lac (mmol/L, $\bar{x}\pm$s)	3.60 ± 3.19	5.00 ± 3.48	2.99 ± 2.70	0.013
ALB (g/L, $\bar{x}\pm$s)	30.7 ± 12.5	27.6 ± 11.4	32.0 ± 12.8	0.022
ALT [U/L, M(P25, P75)]	19.00 (15.00–30.00)	28.60 (22.00–66.00)	17.40 (13.00–22.00)	<0.001
TBIL (μ mol/L, $\bar{x}\pm$s)	14.45 ± 10.67	16.82 ± 16.91	13.43 ± 6.34	0.819
Cr (μmol/L, $\bar{x}\pm$s)	85.38 ± 50.20	115.45 ± 77.01	72.41 ± 23.84	<0.001
cTNI [ng/ml, M(P25, P75)]	0.036 (0.012–0.188)	0.185 (0.040–0.541)	0.020 (0.006–0.055)	<0.001
BNP [ng/L, M(P25, P75)]	151.95 (62.68–340.62)	215.00 (121.02–563.80)	114.25 (56.09–293.52)	0.042
WBC (10^9^/L, $\bar{x}\pm$s)	14.13 ± 6.04	14.35 ± 7.66	14.03 ± 5.28	0.862
HGB (g/L, $\bar{x}\pm$s)	134.63 ± 22.63	133.39 ± 31.49	135.17 ± 17.87	0.805
PLT (10^9^/L, $\bar{x}\pm$s)	181.06 ± 68.98	172.57 ± 75.70	184.72 ± 66.33	0.494
K (mmol/L, $\bar{x}\pm$s)	3.73 ± 0.42	3.77 ± 0.39	3.70 ± 0.43	0.531
Na (mmol/L, $\bar{x}\pm$s)	140.38 ± 5.82	141.78 ± 6.08	139.78 ± 5.66	0.179
Cl (mmol/L, $\bar{x}\pm$s)	107.70 ± 5.31	109.86 ± 5.68	106.77 ± 4.90	0.021
Ca (mmol/L, $\bar{x}\pm$s)	1.96 ± 0.26	1.77 ± 0.30	2.03 ± 0.18	0.001
30-day mortality, *n* (%)	9 (12.3%)	8 (36.4%)	1 (2.0%)	<0.001
Hospitalization length [day, M(P25, P75)]	8 (5–12)	7 (1–12.25)	9 (6–12)	0.175
Total hospitalization costs [Y, M(P25, P75)]	32,214 (17,675–56,588)	52,057 (16,955–76,635)	29,160 (18,314–43,547)	0.115

**FIGURE 3:**
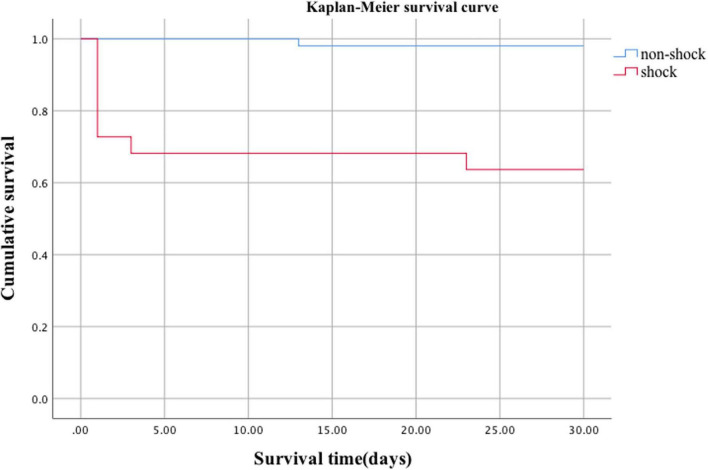
Survival analysis curve of organophosphorus poisoning-induced shock.

The mortality rate of organophosphate poisoning was 12.3% (9/73). The mortality rate in the shock group was significantly higher than that in the non-shock group (36.4 vs. 2.0%, *p* = 0.000) (Table 2). Kaplan–Meier survival analysis showed that the cumulative survival rate of patients in the shock group was significantly lower than that in the non-shock group (Figure 3, Log Rank χ^2^ = 17.653, *p* < 0.001). There was no significant difference between the two groups in the hospital stay and total cost of hospitalization (*p* > 0.05) (Table 2).

Death was considered as the dependent variable, and the Cox proportional hazards regression model was used to screen independent variables with *p* < 0.1 for univariate regression analysis (including shock, Cr, Ca, Cl, and cTNI) (Table 3). Moreover, multivariate COX was performed according to the stepwise method, which showed that shock and Cr were independent risk factors for death (shock: HR, 10.9; 95% CI 1.2–96.3; elevated serum creatinine: HR, 1.0, 95% CI 1.0–1.0) (Table 3).

**TABLE 3:** Cox proportional hazards regression model analysis with multivariate regression of death of organophosphorus poisoning.

Variables	β	SE	HR	95% CI	*P*-value
**Univariate COX regression analysis**					
Shock	2.834	1.068	17.018	2.098–138.063	0.008
Cr	0.011	0.003	1.011	1.006–1.017	0.000
Ca	−2.924	0.991	0.054	0.008–0.375	0.003
Cl	0.140	0.065	1.151	1.014–1.307	0.030
cTNI	0.156	0.080	1.168	0.999–1.366	0.051
**Multivariate COX regression analysis**					
Shock	2.393	1.110	10.946	1.244–96.317	0.031
Cr	0.007	0.003	1.007	1.001–1.014	0.020

PaCO_2_, arterial blood carbon dioxide partial pressure; PaO_2_, arterial partial pressure of oxygen; BE, base excess; Lac, lactic acid; ALB, serum albumin; TBIL, total bilirubin; ALT, alanine aminotransferase; Cr, serum creatinine; AMS, amylase; K, potassium ions; Ca, calcium ions; Na, sodium ions; Cl, chloride ion; cTNI, myocardium troponin; BNP, brain natriuretic peptide; WBC, white blood cell count; HGB, hemoglobin concentration; PLT, the platelet count; PT, prothrombin time; APTT, activate the prothrombin time; Fig, fibrinogen; DD, D dimer; Cr, serum creatinine; AMS, amylase; Ca, calcium ions; Cl, chloride ion; cTNI, myocardium troponin.

## Discussion

Acute organophosphorus pesticide poisoning is a major public health problem in developing countries, especially in Asian countries like China (12, 13). How to reduce mortality in patients with organophosphate poisoning is an urgent problem for clinicians. Shock is one of the main causes of death from organophosphate poisoning and our study found that the mortality rate of patients with acute organophosphate poisoning was 12.3%, and the mortality rate of patients with organophosphate poisoning-induced shock was significantly higher than in the non-shock group (36.4 vs. 2.0%, *p* < 0.001). Kaplan–Meier survival analysis further showed that the cumulative survival rate of patients in the shock group was significantly lower than that in the non-shock group (Log Rank χ^2^ = 17.653, *p* < 0.05). Multivariate regression analysis showed that shock was an independent risk factor for death (HR 10.946, 95%CI 1.244–96.317, *p* = 0.031).Several previous papers have reported hypotension as a significant predictor of death from organophosphate poisoning (5, 8, 14, 15). Based on this, we believe that shock can be used as a reliable early warning indicator of organophosphate poisoning death. Further understanding of the clinical characteristics and unique pathophysiological mechanisms of organophosphate poisoning-induced shock and improving the diagnosis and treatment of it may help to reduce the mortality rate of organophosphate poisoning.

In this study, we attempted to summarize the clinical features of organophosphate poisoning-induced shock. We found that the overall incidence of toxic shock was 30.1%. Kumar Thakur et al. (7) reported that the incidence of 24-h hypotension after organophosphate poisoning was 37.9%, and Dong et al. (14) carried out a retrospective study, which included 871 patients with organophosphate poisoning and found an incidence of hypotension within 24 h of admission of 16.4%; yet the incidence of hypotension varied greatly, which may be related to the difference in the admitted population. In our study, most patients admitted to the emergency department were patients with severe organophosphorus poisoning, which may be the reason for the significantly higher incidence of a shock compared with Dong’s study. We also found that patients with normal blood pressure immediately after admission may progress to shock blood pressure within 24 h of admission. In our study, 40.9% of the shock group developed hypotension at the time of presentation, 27.3% developed hypotension after 6 h, and the longest developed shock blood pressure at 19 h of onset. There are few reports on how long shock occurs after organophosphate poisoning. According to the characteristics of pathophysiological damage by organophosphorus poisoning, shock often occurs in the acute phase 24 h after poisoning (1). In addition, in the early stage of admission, poisoned patients are often accompanied by respiratory failure, hypoxia, other stress states, and even nicotinic symptoms leading to elevated blood pressure, which may contribute to miss of shock. Therefore, when the signs of shock cannot be accurately identified, clinicians need to pay more attention to the 24-h changes in blood pressure and tissue perfusion indicators in patients with organophosphate poisoning. The distribution of dichlorvos in the two groups was also found to be different in our results, with a significantly higher proportion of patients with dichlorvos poisoning in the shock group than in the non-shock group (68.2 vs. 37.3%, *p* < 0.05) and the dose of dichlorvos in the shock group was higher than that in the non-shock group, and it is likely that high doses of dichlorvos poisoning are more likely to result in shock, suggesting that early identification and interrogation of toxic species is also very important in clinical diagnosis and treatment. Lactic acid is considered to have a much higher diagnostic value for shock than hypotension. The Consensus on circulatory shock and hemodynamic monitoring reported by the European Society of Intensive Care Medicine in 2014 (11) and the International Guidelines for Management of Sepsis and Septic Shock in 2016 (16) recommend that lactic acid >2 mmol/L as one of the diagnostic criteria for shock. In our study, the patients in the shock group had higher lactic acid levels; yet, the average lactic acid in the non-shock group was also greater than 2 mmol/L, which may be because of organophosphorus poisoning can cause increased lactic acid by non-perfusion factors. Studies have found that organophosphate poisoning can lead to mitochondrial damage in cells, which in turn may affect the metabolism of lactic acid. In our study, patients in the shock group had higher ALT, cTNI, and Cr. Severe organophosphate poisoning can lead to organ damage. Chen et al. (17) reported that 52% of patients with organophosphate poisoning had elevated cTNI, and organophosphate poisons could directly lead to liver function damage mainly manifested by elevated liver enzymes (18). Organophosphate poisons may also directly damage the glomerulus or renal tubules, leading to increased Cr (19). Besides, shock can also lead to multiple organ dysfunction. Thus, the organs of patients with organophosphate poisoning-induced shock may suffer the double blow of poison and shock, and the organ function will deteriorate. Our results also revealed that the patients in the shock group had significantly lower serum calcium and higher serum chloride. Tripathi et al. (20) found hypocalcemia, hypophosphatemia, and hypomagnesemia in rats with organophosphate poisoning, which may be related to parathyroid hormone. Furthermore, Zhonghua et al. (21) found a significantly increased mortality in patients with hypocalcemia caused by organophosphate poisoning. In our study, the patients in the shock group might have suffered from more severe hypocalcemia due to more severe poisoning. No study reported that organophosphorus poisoning is related to the ion concentration of chloride. However, Suetron et al. (22) found that serum chloride was higher in patients with septic shock and was independently associated with secondary acute kidney injury, which may be caused by the need for infusion with more saline.

We found that elevated Cr immediately after admission was another independent risk factor for death from organophosphate poisoning in addition to shock (HR 1.007, 95%CI 1.001–1.014, *p* = 0.020). In the study of Acikalin et al. (23), elevated Cr was an independent predictor of hospital stay in patients with organophosphate poisoning and an independent predictor of intubation time in mechanically ventilated patients. In addition, Xin et al. (15) found that patients with organophosphate poisoning with elevated Cr also had a higher mortality rate. For patients with organophosphate poisoning-induced shock, the kidneys of patients may suffer double blows from both toxins and shock. Elevated Cr often indicates a poor prognosis. Therefore, elevated Cr during early admission may be an early warning indicator of poor prognosis in patients with organophosphate poisoning.

The unique and complex pathophysiological mechanisms of organophosphorus poisoning-induced shock may account for the fact that shock is an independent risk factor for death. Organophosphorus poisoning can cause hypovolemic shock, cardiogenic shock, and distributive shock (8): (1) Hypovolemic shock: cholinergic crisis, including vomiting, diarrhea, sweating, etc., can lead to loss of body fluids. Hypovolemic shock occurs in cases with severe illness. (2) Cardiogenic shock: cardiogenic shock by organophosphorus poisoning is uncommon, but conventional anti-shock therapy for severe cardiogenic shock is ineffective. Yang et al. (24) reported a case of severe organophosphate poisoning with severe myocardial depression and malignant arrhythmia developed cardiogenic shock and required circulatory support with ECMO. (3) Distributive shock: in 2004, Asari et al. (25) inserted a pulmonary artery catheter in patients with organophosphate poisoning–induced shock and found that the patient’s cardiac output could be maintained, but the peripheral vascular tension significantly decreased. Clinical hemodynamic monitoring supported the occurrence of distributive shock by organophosphate poisoning. There are complex pathophysiological mechanisms in distributive shock caused by organophosphorus poisoning: (1) organophosphorus poisons act on cholinergic receptors in peripheral vascular endothelial cells and cholinergic receptors in the central nervous system, resulting in a comprehensive decrease in peripheral vascular tone (26); (2) organophosphorus poisons may block sympathetic and parasympathetic ganglionic nicotinic transmission, leading to inhibition of baroreflex, and eventually resulting in distributive shock; and (3) organophosphate poisoning may exert mast cell-mediated effects (anaphylaxis) that may lead to histamine-induced hypotension (27). It has been found that high-dose atropine or high-dose vasoactive drugs alone may be difficult to reverse severe distributive shock (10). The pathophysiological mechanism of organophosphorus poisoning shock is complex, and it may be a mixed shock of four types of shocks. There is still no unified and effective therapeutic strategy for organophosphorus poisoning-induced shock, which may be the reason why shock is an independent risk factor for death from organophosphorus poisoning.

This study has a few limitations. First, it is a single-center retrospective study with a small sample size. Clinical information such as GCS scores is unavailable, and there are no data on hemodynamic monitoring for the diagnosis of shock in patients. Subsequent prospective studies with more detailed hemodynamic monitoring in patients with organophosphate poisoning-induced shock are needed.

## Conclusion

This study showed that 30.1% of patients with organophosphate poisoning were diagnosed with shock. Most of them had shock within 24 h. Additionally, we also found the association of the diagnosis of shock and elevated serum creatinine and increased mortality rates in patients with organophosphate poisoning. Given above, the findings of the study also suggested that the diagnosis of shock and elevated serum Cr can be considered as early warning indicators of death in patients with organophosphate poisoning, highlighting the importance of the comprehensive management of shock, especially the control of renal function, in these patients. Finally, our study may provide evidence for clinicians to recognize organophosphate poisoning-induced shock and inform residency training.

## Data availability statement

The original contributions presented in this study are included in the article/Supplementary material, further inquiries can be directed to the corresponding authors.

## Ethics statement

The studies involving human participants were reviewed and approved by Ethics Committee of Fujian Provincial Hospital (Approval number: K 2021-12-069). Written informed consent for participation was not required for this study in accordance with the national legislation and the institutional requirements. Written informed consent was not obtained from the individual(s) for the publication of any potentially identifiable images or data included in this article.

## Author contributions

BX and WZ collected study data and drafted the present manuscript. XS revised the manuscript. All authors read and approved the final version of the manuscript.
